# Prevalence of *Sarcoptes scabiei* Infection in Pet Dogs in Southern China

**DOI:** 10.1155/2014/718590

**Published:** 2014-03-11

**Authors:** Yi-Zhou Chen, Guo-Hua Liu, Hui-Qun Song, Rui-Qing Lin, Ya-Biao Weng, Xing-Quan Zhu

**Affiliations:** ^1^College of Veterinary Medicine, South China Agricultural University, Guangzhou, Guangdong 510642, China; ^2^State Key Laboratory of Veterinary Etiological Biology, Key Laboratory of Veterinary Parasitology of Gansu Province, Lanzhou Veterinary Research Institute, Chinese Academy of Agricultural Sciences, Lanzhou, Gansu 730046, China; ^3^College of Veterinary Medicine, Hunan Agricultural University, Changsha, Hunan 410128, China

## Abstract

Little is known about the prevalence of *Sarcoptes scabiei* infection in pet dogs in China. In the present study, the prevalence of *S. scabiei* infection in pet dogs in Guangzhou, southern China, was investigated between January and December, 2009. A total of 3,977 pet dogs admitted to animal hospitals were examined for the presence of *S. scabiei* using a parasitological approach. The average prevalence of *S. scabiei* infection in pet dogs is 1.18% (95% confidence interval (CI): 0.85–1.52%). The prevalence of *S. scabiei* was higher in winter (1.42%; 95% CI: 0.29–2.55%), summer (1.39%; 95% CI: 0.83–1.96%), and autumn (1.1%; 95% CI: 0.53–1.68%) than in spring (0.63%; 95% CI: 0.02–1.25%). Furthermore, the prevalence of *S. scabiei* was the highest in Pekingese (21.88%; 95% CI: 7.55–36.2%), followed by Papillon (5.26%; 95% CI: 0–11.06%) and Bichon Frise (3.19%; 95% CI: 0–6.75%). The results of the present investigation indicate that *S. scabiei* infection is prevalent in pet dogs in Guangzhou, China, which provides relevant “baseline” data for conducting control strategies and measures against scabies in this region and elsewhere in China. To our knowledge, this is the first comprehensive report of *S. scabiei* prevalence in pet dogs in China.

## 1. Introduction

Scabies is an emerging or reemerging infectious disease caused by the mite* Sarcoptes scabiei* that threatens globally human and animal health [1]. It is estimated that about 300 million people worldwide are currently infected with* S. scabiei* [2]. There is a general agreement that* S. scabiei* from humans and animals represents a single species [3, 4].

*S. scabiei* can also infect animal hosts, including cat [5], giraffe [6], pig [7], raccoon dog [8], rabbit [9], sheep [10], serow [11], and wolf [12], leading to major economic losses [13]. Scabies is a major problem in dogs, for example, approximately 20% of dogs in some regions of the Korea experiencing* S. scabiei* [14]. The mite can invade many different body parts of dogs and can cause erythema, papules, lichenification, scales, crusts, and alopecia [15].

Although the prevalence of* S. scabiei* infection in pet dogs has been reported in some countries [16, 17], little is known about the prevalence of* S. scabiei* infection in pet dogs in China [18–20]. Moreover, these preliminary pilot surveys showed that* S. scabiei* is highly prevalent in pet dogs in China. Therefore, the objective of the present investigation was to examine the* S. scabiei* prevalence in pet dogs in Guangzhou, southern China. The results should provide a foundation for the control of* S. scabiei* infection in pet dogs in this region and elsewhere in China.

## 2. Materials and Methods

### 2.1. Examination of Pet Dogs for the Presence of* S. scabiei* and Data Collection

From January to December, 2009, a total of 3,977 pet dogs admitted to animal hospitals in Guangzhou, Guangdong province, southern China, were examined for the presence of* S. scabiei* (Table 1). Before sampling, pet dogs were subjected to clinical examination to determine their health status. Information about each pet dog, such as age, medical history, sex, breed, and weight, was collected. All pet dogs, from which* S. scabiei* were examined, were handled in strict accordance with good animal practice as defined by the relevant national and/or local animal welfare bodies, and all animal work was approved by the appropriate committee. The presence of* S. scabiei* was detected by microscopic examination of deep skin scraping, plucked hairs, and skin biopsy. Identification of* S. scabiei* was conducted by morphological criteria and site of predilection [13].

### 2.2. Statistical Analysis

The data were analyzed statistically using the PASW Statistics 18 (IBM Corporation, Somers, NY, USA); 95 % confidence intervals (CI) are given. The value of *P* < 0.05 differences between levels within factors and interactions was considered to be statistically significant.

## 3. Results and Discussion

The overall prevalence of* S. scabiei* in pet dogs in Guangzhou, southern China, was 1.18% (95% CI: 0.85–1.52%) (Table 1). The prevalence in female pet dogs (1.23%) was slightly higher than that in male pet dogs (1.15%) (Table 1). The* S. scabiei* prevalence was higher (*P* > 0.05) in winter (1.42%), summer (1.39%), and autumn (1.1%) than in spring (0.63%) (Table 1). The prevalence of* S. scabiei* in pet dogs of less than 1 year old (1.15%) was higher than in pet dogs of other age groups (Table 2). Furthermore, the prevalence of* S. scabiei* was the highest in Pekingese (21.88%), followed by Papillon (5.26%) and Bichon Frise (3.19%) (not shown).

Scabies is a significant public health problem and causes considerable economic impact on livestock industry around the world [21]. The present study provides the first comprehensive assessment of* S. scabiei* infection in pet dogs in Guangzhou, southern China. The present survey showed that the* S. scabiei* prevalence in pet dogs was 1.18% (95% CI: 0.85–1.52%), which was lower than that reported in Nigeria (2.0%) [16] and Iran (5.56%) [17]. These differences may be related to climate conditions, such as humidity and temperature, as well as the susceptibility of different breeds of dogs.

The present investigation showed that prevalence of* S. scabiei* is the highest in winter (1.42%; 95% CI: 0.29–2.55%), followed by summer (1.39%; 95% CI: 0.83–1.96%), and was the lowest in spring (0.63%; 95% CI: 0.02–1.25%). These results suggest that* S. scabiei* is prevalent all year round, with the peaks in winter (cold season) and summer (moist season), which is consistent with that of a previous study [22]. Cold weather encourages increased physical crowding of pet dogs and* S. scabiei* can survive longer away from the host in lower temperatures [2]. Furthermore, the more frequent incidence in moist season might be because these conditions are favorable for mite reproduction.

The present study revealed that the prevalence of* S. scabiei* in pet dogs of less than 1 year old was higher than in pet dogs of other age groups, suggesting that young pet dogs appear to be more susceptible to* S. scabiei* than adult pet dogs. This is most likely due to their constant exposure to carrier mothers/owners because scabies is transmitted by direct person-to-person body contact. Furthermore, the prevalence of* S. scabiei* was the highest in Pekingese (21.88%; 7/32) although it is not popular as pet dogs in China. However, high prevalence in Pekingese poses a significant health risk for humans because the dog scabies can be transmitted to humans [23, 24].

## 4. Conclusion

In summary, the results of the present survey indicate that* S. scabiei* infection is prevalent in pet dogs in Guangzhou, southern China, but this severe situation has received little attention in the past. Therefore, it is imperative to take integrated control strategies and measures to prevent and control* S. scabiei* infection in pet dogs in this region and elsewhere in China. To our knowledge, this is the first comprehensive report of* S. scabiei* prevalence in pet dogs in China.

## Figures and Tables

**Table 1:** Seasonal prevalence of *Sarcoptes scabiei* in pet dogs in Guangzhou, southern China.

Season	No. examined	No. positive	Prevalence (%)
Spring	633	4	0.63
Summer	1652	23	1.39
Autumn	1269	14	1.1
Winter	423	6	1.42

Total	3977	47	1.18

**Table 2:** Prevalence of *Sarcoptes scabiei* infection in pet dogs of different age groups in Guangzhou, southern China.

Age	No. examined	No. positive	Prevalence (%)
<1 yr	2007	27	1.35
1–5 yr	1486	16	1.08
>5 yr	484	4	0.83

Total	3977	47	1.18
